# Bleeding sites and treatment strategies for cardiac tamponade by catheter ablation requiring thoracotomy: risks of catheter ablation in patients with left atrial diverticulum

**DOI:** 10.1186/s13019-024-02710-1

**Published:** 2024-04-17

**Authors:** Atsuyuki Mitsuishi, Yujiro Miura, Yoshinori Nomura, Takayoshi Hirota, Naoki Arima, Hiroaki Kitaoka, Hiroki Tateiwa, Yoshifumi Katsumata

**Affiliations:** 1 Department of Cardiovascular Surgery, Kochi Medical School Hospital, 185-1, Kohasu, Okohmachi, Nankoku-shi, Kochi Prefecture 783-8505 Japan; 2 Department of Clinical Engineering, Kochi Medical School Hospital, 185-1, Kohasu, Okohcho, Nankoku-shi, Kochi Prefecture 783-8505 Japan; 3 Department of Cardiology and Geriatrics, Kochi Medical School Hospital, 185-1, Kohasu, Okohmachi, Nankoku-shi, Kochi Prefecture 783-8505 Japan; 4 Department of Anesthesiology and Intensive Care Medicine, Kochi Medical School, 185-1, Kohasu, Okohmachi, Nankoku-shi, Kochi Prefecture 783-8505 Japan

**Keywords:** Cardiac tamponade, Catheter ablation, Atrial fibrillation, Left atrial diverticulum, Venoarterial extracorporeal membrane oxygenation, Case series

## Abstract

**Background:**

There is insufficient information regarding the bleeding sites and surgical strategies of cardiac tamponade during catheter ablation for atrial fibrillation (AF).

**Case presentation:**

Of the five patients with cardiac tamponade, three required surgical intervention and two required pericardiocentesis. In the first case of three cardiac tamponades requiring surgical intervention, considering that the peripheral route was used, the catecholamines did not reach the heart, and due to unstable vital signs, venoarterial extracorporeal membrane oxygenation (VA-ECMO) was inserted. No bleeding point was identified, but a thrombus had spread around the left atrium (LA) with diverticulum. Hemostasis was achieved with adhesives placed around the LA under on-pump beating. In the second case, pericardiocentesis was performed, but the patient showed heavy bleeding and unstable vital signs. Thus, VA-ECMO was inserted. Heavy bleeding was expected, and safety was enhanced by attaching a reservoir to the VA-ECMO. The bleeding point was found between the left upper pulmonary artery and LA under cardiac arrest to obtain a good surgical view for suturing repair. In the third case, the LA diverticulum was damaged. Pericardiocentesis resulted in stable vitals, but sustained bleeding was present. A bleeding point was found at the LA diverticulum, and suture repair under on-pump beating was performed.

**Conclusions:**

When cardiac tamponade occured in any patient with LA diverticulum, treatment could not be completed with pericardiocentesis alone, and thoracotomy was likely to be necessary. If the bleeding point could be confirmed, suturing technique is a more reliable surgical strategy than adhesive alone that leads to pseudoaneurysm. If the bleeding point is unclear, it is important to confirm the occurrence of LA diverticulum using a preoperative CT, and if confirmed, cover it with adhesive due to a high possibility of diverticulum bleeding. The necessity of CPB should be determined based on whether these operations can be completed while maintaining vital stability.

**Supplementary Information:**

The online version contains supplementary material available at 10.1186/s13019-024-02710-1.

## Background

Catheter ablation for atrial fibrillation (AF) is increasingly used. Although the safety of catheter ablation has improved owing to technological innovation, many cases of cardiac tamponade as a fatal complication have been reported [1]. However, information regarding the site of bleeding and surgical strategy is still lacking.

## Case presentation

### Patient 1

**Fig. 1:**
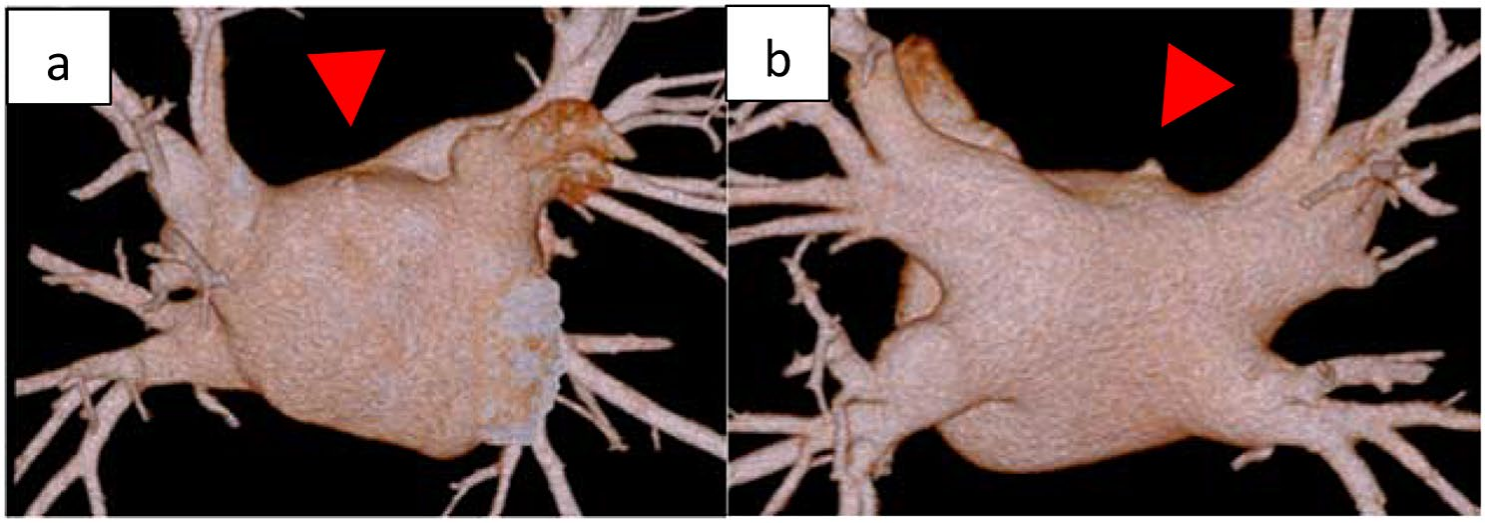
Case 1 **a**, **b**: Perioperative computed tomography showing a left atrial diverticulum (red arrow). **a**: Anterior left atrium, **b**: Posterior left atrium

Case 1 was a 68-year-old man diagnosed with tachycardiac AF 6 months earlier and was treated with direct oral anticoagulant, followed by ablation. During the procedure, an atrial septal puncture was performed while confirming intracardiac ultrasound (ICE), and left atrial angiography was performed during continuous right ventricular pacing (160 ppm). During pacing from the coronary sinus, a mapping catheter (HD grid®, Abbot, Illinois, United States) was used to map the left atrium (LA), followed by bilateral pulmonary vein isolation, posterior wall isolation, and superior vena cava isolation. After the superior vena cava was isolated, pericardial effusion was suspected on fluoroscopic imaging and cardiac tamponade was diagnosed by intracardiac echocardiography. Although pericardiocentesis was performed from the epigastric region, the circulation was unstable, and the response to vasopressors which was administered peripherally was poor. A venoarterial extracorporeal membrane oxygenation (VA-ECMO) was inserted from the right inguinal region, and emergent surgery was performed. Median sternotomy was used to perform a longitudinal pericardial incision and aspirate the blood carefully. Then, no oozing blood was observed. Cardiopulmonary bypass (CPB) was established by cannulation on the ascending aorta and the right atrium. A hematoma was found around the LA, but the bleeding point could not be identified after intrapericardial lavage. Bleeding from the LA diverticulum, observed preoperatively (Fig. 1), was suspected, but could not be identified during surgery. The LA was sealed using Tacoseal® (Tacoseal® tissue adhesive sheet, CSL Behring Co., Ltd., Japan). The VA-ECMO inserted in the right inguinal region was removed, and the operation was completed.

### Patient 2

**Fig. 2:**
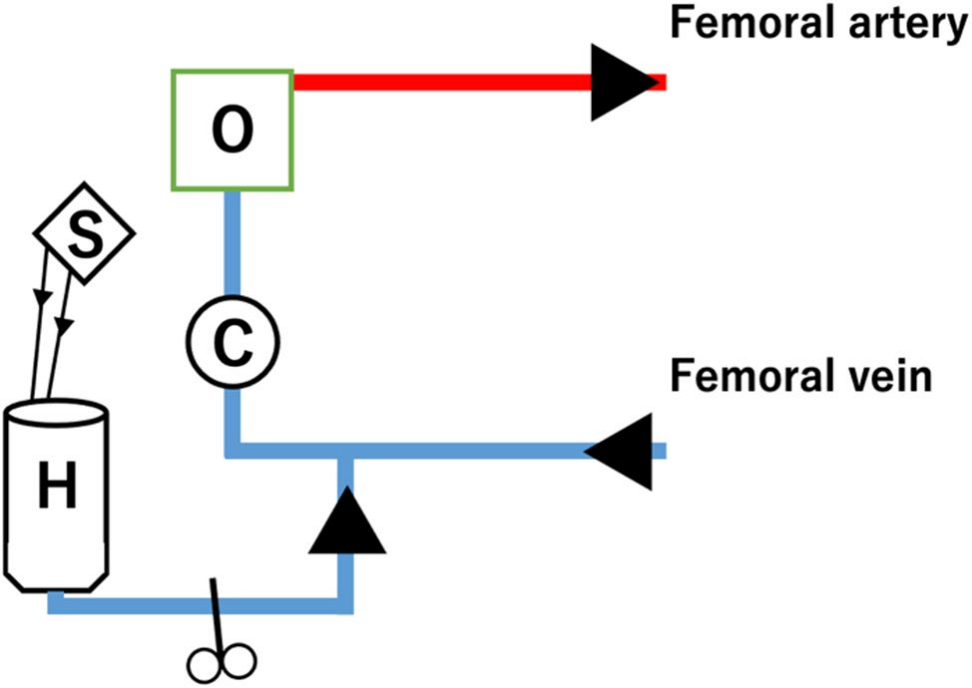
Attachment of a reservoir to a venoarterial extracorporeal membrane oxygenation. O:Membrane oxygenator, C:Centrifugal pump, H:Hard shell reservoir, S:Suction (blood with heparinized saline solution)

**Fig. 3:**
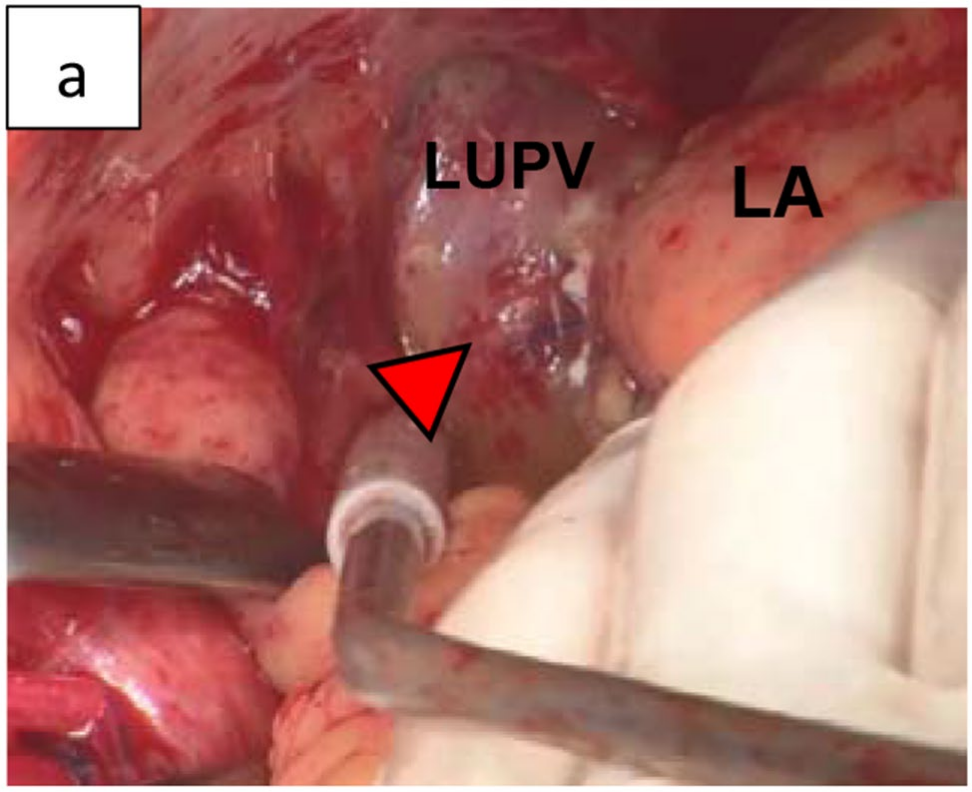
Case 2. Bleeding point between the left upper pulmonary vein and left atrium

Case 2 was a 73-year-old man who was aware of AF 3 months ago and took bisoprolol orally, but the subjective symptoms did not improve, and ablation was decided. During procedure, an atrial septal puncture was performed while confirming ICE, and left atrial angiography was performed during continuous right ventricular pacing (180 bpm). Intracardiac cardioversion (5 J) was performed, and the LA was mapped using a mapping catheter (HD grid®) during coronary sinus pacing. Electricity was started from the left lower pulmonary vein, and blood pressure decreased while electrifying the anterior wall of the left pulmonary vein. Cardiac tamponade was diagnosed based on cardiac imaging and intracardiac echocardiography findings. Although pericardiocentesis was performed, the blood pressure did not rise. A cardiac massage was started, and vasopressors were administered. Since the blood transfusion was not in time, the drained blood was returned to the body through the sheath of the femoral vein to maintain the circulating blood volume. Blood pressure was maintained with VA-ECMO, but bleeding from pericardial drainage did not stop, so it was determined that open heart surgery was necessary. Heavy bleeding was expected, and safety was enhanced by attaching a reservoir to the VA-ECMO (Fig. 2). During surgery, median sternotomy and partial incision of the pericardium led to continuous bloody pericardial effusion. A pericardial incision was performed, and persistent bleeding from the left side of the heart was confirmed. CPB was established under stable conditions under VA-ECMO with cannulations on ascending blood and right atrium. Then, VA-ECMO was completed. After confirmation of a longitudinal laceration of approximately 5 mm at the transition to the left upper pulmonary vein and LA (Fig. 3) and bleeding from the same site, it was determined that treatment under cardiac arrest was necessary to obtain sufficient surgical view for suturing repair. An antegrade cardioplegic solution was injected for cardiac arrest. Hemostasis was achieved by mattress suturing using 4 − 0 polypropylene with pledget.

Ablation was incomplete and LA appendage resection was performed by mattress suturing using 4 − 0 polypropylene with pledget.VA-ECMO was removed, and the operation was completed.

### Patient 3

**Fig. 4:**
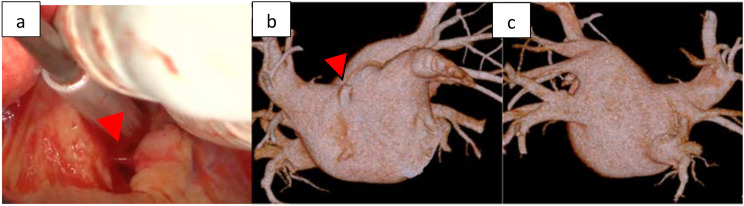
Case 3. **a**: Bleeding point of a left atrium diverticulum. **b**,**c**: Perioperative computed tomography showing a left atrial diverticulum (red arrow). **b**: Anterior left atrium, **c**: Posterior atrium

Case 3 was a 59-year-old man who had paroxysmal AF 1 year earlier and started regular oral administration of pilsicainide. However, after that, palpitations were still observed, and the frequency was increasing, so ablation was decided. During procedure, an atrial septal puncture was performed while confirming ICE, and left atrial angiography was performed during continuous right ventricular pacing (180 bpm). A mapping catheter (HD grid®) was used to map the LA during coronary sinus pacing. Electricity was started from the left lower pulmonary vein, and blood pressure decreased while conducting electricity from the upper wall side of the left pulmonary vein. Cardiac tamponade was diagnosed based on the findings of cardiac imaging and intracardiac echocardiography. Given that it was difficult to maintain blood pressure with intravenous fluids, pericardiocentesis was performed from the epigastric region. By aspirating the pericardial fluid, we were able to maintain a low blood pressure but sustained bleeding of ≥ 300 ml for 1 h was present. We considered spontaneous hemostasis difficult; thus, we decided to perform surgical hemostasis by thoracotomy. During surgery, median sternotomy and partial incision of the pericardium led to continuous bloody pericardial effusion. A pericardial incision was performed, and persistent bleeding from the left side of the heart was confirmed. CPB was established by cannulation on ascending blood and right atrium. Persistent bleeding was observed from the LA (Fig. 4a) [see Additional file 1]. It was consistent with the diverticulum (Fig. 4b, c) located on the septal side of the right pulmonary vein, confirmed by cardiac CT and was judged to be bleeding from the same site. Hemostasis was achieved by repair using 4 − 0 polypropylene with pledget using the on-pump beating technique.

No complications, such as postoperative infection (including mediastinitis), cerebrovascular disease, or renal dysfunction were observed in any of the three patients. Cases 1 and 3 where ablation was completed were discharged home with sinus rhythm, and case 2 where ablation treatment was insufficient and LA appendage resection was added was discharged with AF.

## Discussion and conclusions

Among the complications associated with catheter ablation, cardiac tamponade is the most feared. Its incidence varies from 0.3 to 2.5% [2–4]. In this study, among 1,083 catheter ablation cases for AF, we encountered five cardiac tamponades (0.5%; 5 of 1083). Of the five patients with cardiac tamponade, three required sternotomy and two required pericardiocentesis (Table 1). Although one of five in our study were woman, the incidence of cardiac tamponade due to catheter ablation was reported to be significantly higher in women. The women’s hearts are smaller than those of men’s, but the same catheters are used in both sexes. This increases the groin complication rate at the puncture site and also increases the risk of perforation and transseptal mispuncture [5]. The incidence of atrial septal aneurysm is reported to be higher in women, and performing transseptal puncture may be more difficult in women than in men. Furthermore, most cases of cardiac tamponade occur during catheterization or ablation due to the thin LA wall and small atrial volume in women [6]. Owing to the small number of analyzed cases in the present study, we were unable to demonstrate a direct relationship between sex and the risk of cardiac tamponade.

**Table 1:** Summary of cases with sternotomy and pericardiocentesis

	Gender	Year	Rupture Point	Cardiac arrest	Treatment	LA diverticulum
Case 1	Male	63	Not identify (Hematoma around LA)	On-pump beating	Adhesive	Yes
Case 2	Male	78	Between the left upper pulmonary vein and LA	Cardiac arrest	Suturing	No
Case 3	Male	59	LA diverticulum	On-pump beating	Suturing	Yes
Case 4	Female	65	Not identify	None	Pericardiocentesis	Yes
Case 5	Male	64	Not identify	None	Pericardiocentesis	Yes

LA: Left atrium

Interestingly, cases 1 and 3 had LA diverticulum, and both patients who were treated only by pericardiocentesis also had LA diverticulum. The frequency of LA diverticulum was 36.0% in patients with AF [7]. In this study, 80% (4 of 5) of patients with cardiac tamponade by catheter ablation had LA diverticulum, with a high frequency. Diagnosis with LA diverticulum can be delayed until adulthood because most of them are asymptomatic. Surgical resection of LA diverticulum was considered appropriate because of the patient’s symptom of compression and the risk of thrombosis and rupture [8]. LA diverticulum may be a source for ectopic arrhythmogenic foci [9]. The frequency of complications in ablation therapy for AF may be higher in patients with LA diverticulum than those without such conditions. It is believed that low blood flow in the diverticulum promotes excessive heating at low power, increasing the risk of vapor rupture and coagulum formation. Moreover, if the LA diverticulum is long, the catheter tip could become trapped. The wall of the LA diverticulum was much thinner than that of the adjacent LA, resulting in a potentially vulnerable area for perforation in LA during radiofrequency ablation. The most common location for left atrial diverticula is the right anterior–superior wall of the LA [9]. The close vicinity between the orifice of the LA diverticulum and common ablation sites, including the ostia of adjacent pulmonary veins and the LA appendage might increase the possibility of contact between the ablation catheter and LA diverticula [10]. Even if the LA diverticulum does not come out in line with the ablation line, it is possible that the ablation catheter tip erroneously entered this diverticulum, causing damage.

The treatment methods for cardiac tamponade are broadly classified into four categories: conservative treatment, pericardiocentesis, interventional treatment with a closure device [7], and surgical treatment. A combination of subxiphoid pericardial puncture and anticoagulation is appropriate for the initial treatment of cardiac tamponade in AF ablation, although definitive management may require surgical intervention [11]. The rate of surgical treatment after pericardiocentesis was reported by Michowitz et al. [6]. Tamponade occurred in 0.9% of 34,943 patients undergoing AF ablation, which was treated by pericardiocentesis in 84% of the patients, surgery followed by pericardiocentesis in 15%, and surgery in 1%. Surgery was performed on the patient who did not undergo pericardiocentesis. Thus, in many cases, cardiac tamponade after catheter ablation can be stabilized by pericardial puncture and conservative treatment. This is thought to be due to the spontaneous sealing of the cardiac perforation caused by the catheter, presumably to achieve drainage and hemostasis within the pericardial space [12]. According to these references, the incidence of cardiac tamponade in this study was 0.46%, which is quite low. However, the surgical intervention rates are high (three of five; 60%). In fact, case 1 required surgical treatment even though no active bleeding was observed. This is thought to be due to the delay in the delivery of the vasopressor, which was administered peripherally when cardiac massage was required, leading to unstable circulation and the insertion of VA-ECMO and surgical treatment. Generally, whether pericardiocentesis can provide adequate therapy depends on the severity of the primary injury, presence or absence of complications during the pericardiocentesis, promptness of treatment, and patient’s overall cardiovascular reserve [11]. Surgical treatment is then considered in cases of hemodynamic instability or inadequate drainage despite pericardial puncture. Surgical treatments include thrombectomy with or without CPB [13, 14], suturing, application of adhesives such as Tachoseal® [15, 16], and their combinations. Additionally, if the perforation site is fragile or very close to the coronary artery, applying adhesive alone without suturing may be considered, but there have been reports of cases in which pseudoaneurysms later developed at the perforation site [17]. Postoperative follow-up is also important. Adhesives were used in case 1, but no pseudoaneurysm was observed 3 years after the operation. Regarding the necessity of CPB, it should be considered when CPB is indispensable for the purpose of bleeding control and cardiac arrest securing the visual field such as to ensure hemostasis around the LA appendage, as in this case. Additionally, there are also reports of bleeding control by attaching a reservoir under the use of VA-ECMO [18]. When VA-ECMO is already attached and active bleeding is expected, thoracotomy can be safely performed by attaching a reservoir to VA-ECMO for blood-loss management as in case 2 (Fig. 2).

Out of 1,083 catheter ablation cases for AF, we encountered five cardiac tamponades during our experiment (Table 1). We observed that four of five patients had LA diverticulum, which can increase the risk for cardiac tamponade during catheter ablation for AF. The catheter may become entangled or/and be easy to perforate LA diverticulum, leading to cardiac tamponade. It is difficult to view the entire LA, and there is a high possibility of overlooking the bleeding point from LA. In particular, when the bleeding site is not clearly detected in patients with LA diverticulum, the area around the LA diverticulum should be carefully searched.

Futhermore, when cardiac tamponade occured in any patient with LA diverticulum, treatment could not be completed with pericardiocentesis alone, and thoracotomy was likely to be necessary. If the bleeding point could be confirmed, suturing technique is a more reliable surgical strategy than adhesive alone, leading to pseudoaneurysm. If the bleeding point is unclear, it is important to confirm the occurrence of LA diverticulum using a preoperative CT, and if confirmed, cover it with adhesive due to a high possibility of diverticulum bleeding. The necessity of CPB should be determined based on whether these operations can be completed while maintaining vital stability.

Due to the retrospective nature of the study, it remains uncertain whether LA diverticula are associated with increased cardiac tamponade via catheter ablation for AF. Thus, further studies are warranted in the future.

### Electronic supplementary material

Below is the link to the electronic supplementary material.

**Additional file 1**: Case 3; Bleeding point of a left atrium diverticulum

## Data Availability

Not applicable.

## Abbreviations

AF: Atrial fibrillation
ICE: intracardiac ultrasound
LA: Left atrium
VA-ECMO: venoarterial extracorporeal membrane oxygenation
CPB: Cardiopulmonary bypass
